# Assessment of proximal tibial fractures with 3D FRACTURE (fast field echo resembling a CT using restricted echo-spacing) MRI—intra-individual comparison with CT

**DOI:** 10.1007/s00330-025-11522-3

**Published:** 2025-03-24

**Authors:** Inka Ristow, Shuo Zhang, Christoph Riedel, Alexander Lenz, Ralph Akoto, Matthias Krause, Gerhard Adam, Peter Bannas, Frank Oliver Henes, Lennart Well

**Affiliations:** 1 Department of Diagnostic and Interventional Radiology and Nuclear Medicine, University Medical Center Hamburg-Eppendorf, Hamburg, Germany; 2 Philips, Best, The Netherlands; 3 Department of Trauma Surgery, Orthopaedics and Sports Traumatology, BG Hospital Hamburg, Hamburg, Germany; 4 Department of Trauma and Orthopaedic Surgery, University Medical Center Hamburg-Eppendorf, Hamburg, Germany; 5 Department of Diagnostic and Interventional Radiology, BG Hospital Hamburg, Hamburg, Germany

**Keywords:** Bone, Contrast, Magnetic resonance imaging, Computed tomography, Tibial fracture

## Abstract

**Objectives:**

To evaluate the feasibility and diagnostic performance of a 3D FRACTURE (fast field echo resembling a CT using restricted echo-spacing) MRI sequence for the detection and classification of proximal tibial fractures compared with CT.

**Methods:**

We retrospectively included 126 patients (85 male; 39.6 ± 14.5 years) from two centers following acute knee injury. Patients underwent knee MRI at 3 T including FRACTURE-MRI. Additional CT was performed in patients with tibial fractures (32.5%; *n* = 41) as the reference standard for fracture classification. Two radiologists independently evaluated FRACTURE-MRI for the presence of fractures and classified them according to AO/OTA, Schatzker, and the 10-segment classification. Diagnostic performance of FRACTURE-MRI was assessed using crosstabulations. Inter-reader agreement was estimated using Krippendorff’s alpha. Image quality was graded on a five-point scale (5 = excellent; 1 = inadequate definition of fracture lines and fracture displacement) and assessed using estimated marginal means.

**Results:**

Fractures were detected by FRACTURE-MRI with a sensitivity of 91.5% (83.2–96.5%) and a specificity of 97.1% (93.3–99.0%). Regarding fracture classification, diagnostic performances were slightly lower, with the 10-segment classification yielding the best sensitivity of 85.7% (81.4–89.3%) and specificity of 97.4% (96.6–98.0%), and the Schatzker classification yielding the lowest sensitivity of 78.2% (67.4–86.8%) and specificity of 97.7% (94.1–99.4%). Inter-reader agreement across the whole cohort was excellent (Krippendorff’s alpha 0.89–0.96) and when considering only patients with fractures, good to acceptable (0.48–0.91). Image quality was rated good (estimated marginal mean 4.3 (4.1–4.4)).

**Conclusion:**

FRACTURE-MRI is feasible at 3 T enabling accurate delineation of fracture lines for precise diagnosis and classification of proximal tibial fractures.

**Key Points:**

***Question*** *CT-like MRI is increasingly being evaluated for its advantages in bone imaging but is not yet established in routine practice*.

***Findings*** *The 3D FRACTURE (fast field echo resembling a CT using restricted echo-spacing) MRI sequence is feasible at 3 T, allowing for diagnosis and classification of proximal tibial fractures*.

***Clinical relevance*** *FRACTURE-MRI might be a helpful alternative to computed tomography in an acute trauma setting by reducing costs and radiation exposure in patients requiring a preoperative MRI anyway.*

**Graphical Abstract:**

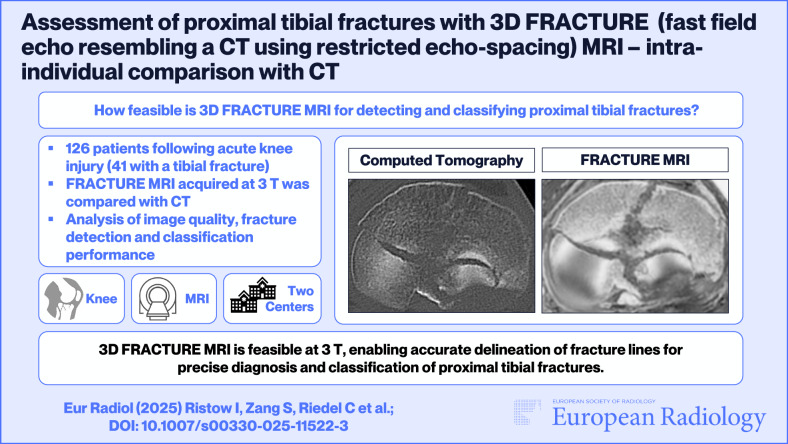

## Introduction

Proximal tibial fractures account for 1.2% of all fractures, with an incidence of about 13 per 100,000 [1]. Tibial fractures are typically a result of high-energy trauma in young patients and low-energy falls in older patients [2], with severities ranging from nondisplaced to complex fractures with intraarticular involvement. Due to their complexity and risk of secondary post-traumatic arthritis, including chronic pain and instability, they pose considerable challenges in clinical treatment [3–5].

In addition to conventional radiography, three-dimensional computed tomography (CT) is the diagnostic gold standard to precisely visualize the skeletal structures, to accurately assess the fracture type, and to consecutively determine the best individual treatment strategy. Moreover, it can easily be combined with angiography in case of suspected vascular injury involving the popliteal artery. However, CT is associated with radiation exposure.

Increased availability of CT scanners has contributed to a better understanding of the underlying trauma mechanism, resulting in the development of numerous fracture classifications based thereon to facilitate clinical decision-making [6]. The classifications according to Schatzker et al [7], the AO/OTA system (Arbeitsgemeinschaft für Osteosynthesefragen/Orthopedic Trauma Association, [8]), Moore et al [9], and the 10-segment classification by Krause et al [10] are widely used classifications to distinguish between extraarticular fractures, simple split fractures, and complex split- and/or depression-fractures involving either one or both tibial condyles.

In addition to CT, magnetic resonance imaging (MRI) as an ionization radiation-free cross-sectional imaging technique with excellent soft tissue contrast is recommended to detect possible ligamentous, meniscal, and/or muscular concomitant injuries [11]. However, one of the major disadvantages of MRI is insufficient visualization of the bone due to its very short T2/T2* relaxation time. While this can be mitigated by using ultrashort (UTE) or zero (ZTE) echo time sequences [12, 13], they rely on specific pulse sequences and hardware adoption. On the other hand, gradient echo sequences (GRE) for “black-bone” or susceptibility-weighted imaging (SWI) are widely available independent of vendors or field strengths to visualize the skeletal structures. FRACTURE (fast field echo resembling a CT using restricted echo-spacing) is a recently introduced MRI technique based on 3D multi-echo GRE that has shown excellent cortical and trabecular bone contrast yielding clinically relevant information for disease diagnosis and management in a cohort of pediatric patients [14].

The aim of this study was to evaluate the diagnostic performance of the CT-like 3D FRACTURE-MRI in a large group of adult patients for the detection and classification of proximal tibial fractures following acute knee injury in a two-center setup.

## Methods

### Study design

The retrospective study was approved by the local institutional review board (Ärztekammer Hamburg, 2024-300434-WF). Informed consent of patients was waived due to retrospective analysis of anonymized data.

**Fig. 1:**
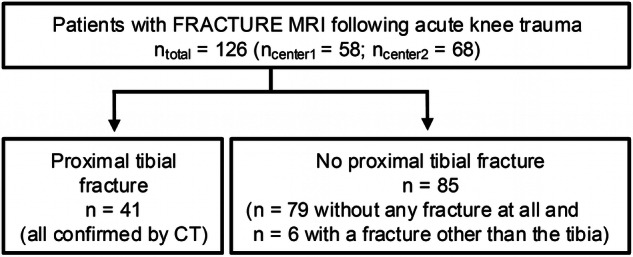
Flowchart illustrating the patient selection process

In this two-center study, all patients following acute knee injury who presented to the emergency departments between 05/2022 and 03/2024 and who underwent subsequent MRI of the knee at 3 T including FRACTURE-MRI were included (*n* = 126; Fig. 1). An additional CT scan was performed in patients with acute fractures (*n* = 41). Reference standard regarding the presence or absence of a proximal tibial fracture was determined under consideration of the complete MRI study including fluid-sensitive sequences, conventional radiographs of the knee, and the CT scan in patients with acute fractures.

### MRI data acquisition

MR imaging was performed at 3 T as part of the clinical routine (both centers: Philips Ingenia) using a dedicated 16-channel knee coil with maximum knee flexion of 10°. Standard imaging protocols included a localizer, axial, coronal, and sagittal fat-saturated proton density-weighted sequences, and a coronal T1-weighted sequence followed by the proposed FRACTURE-MRI sequence. The detailed scan parameters of the FRACTURE-MRI technique at both centers are reported in Table 1. For image evaluation, 3D FRACTURE-MRI was reformatted in sagittal, coronal, and axial orientations, inverted grayscales, and windowing resembling a CT-like bone window.

**Table 1:** MR image parameters used in the two different centers

	Center 1	Center 2
Basic pulse sequence	3D FFE	3D FFE
FOV (mm^2^)	130 × 160	160 × 160
Matrix ACQ	144 × 174	180 × 180
Voxel ACQ (mm^3^)	0.90 × 0.92 × 0.90	0.90 × 0.90 × 0.90
Voxel REC (mm^3^)	0.66 × 0.66 × 0.75	0.63 × 0.63 × 0.45
TR / TE / delta TE (ms)	23 / 4.6 and 5.0	4.2 / 1.86
SENSE or C-SENSE	C-SENSE 5.5	SENSE 2 × 1
Scan time (min)	3:21	4:22
Scan orientation	Axial	Sagittal
MPR	Coronal, Sagittal	Axial, Coronal

*FFE* fast field echo, *SENSE* sensitivity encoding, *C-SENSE/CS* compressed SENSE, *FOV* field of view, *ACQ* acquired, *REC* reconstructed, *TR* repetition time, *TE* echo time, *MPR* multiplanar reconstruction

### CT data acquisition

CT images were acquired as part of the clinical routine in patients with acute fractures. In center 1, imaging was performed using a SOMATOM Force scanner (Siemens Healthineers) with the following scan parameters: 100 kV; pitch 0.80; collimation 0.6 mm; reconstructed slice thickness 1 and 3 mm. In center 2, imaging was performed using a Toshiba Aquilion scanner (Canon Medical Systems) with the following scan parameters: 120 kV; pitch 0.64; collimation 0.5 mm; reconstructed slice thickness 2 mm. A bone-specific convolution kernel for image reconstruction in axial, coronal, and sagittal orientation was applied at both centers.

### Data analysis

#### Reference standard

All scans were reviewed using the institutional picture archiving and communications system (PACS, Centricity Universal Viewer, GE Healthcare).

Patients were classified as fracture-negative if neither conventional radiographs of the knee nor the complete clinical MRI examination (including fluid-sensitive sequences) showed any evidence of bone trauma.

Patients were classified as fracture-positive if a fracture could be identified on either the conventional X-ray images and/or the complete clinical MRI examination. In case of an identified fracture, an additional CT scan was performed, serving as the diagnostic reference standard for fracture classification. Classification of the fracture type according to AO/OTA, Schatzker, and the 10-segment classification was defined in consensus by three radiologists (I.R., L.W., F.O.H.) with 5, 10, and 17 years of experience and one trauma surgeon (M.K.) with 11 years of experience based on the CT scans. To reach a common agreement on how to correctly classify the tibial fractures, the CT-based consensus reading was carried out before the actual FRACTURE-MRI evaluation. Schematic illustrations of the different classifications from the original papers were used to facilitate decision-making.

#### Diagnostic performance

FRACTURE-MRI scans were independently evaluated by two radiologists (I.R., L.W.) with 5 and 10 years of experience. The time interval between the definition of the CT-based diagnostic reference standard and the FRACTURE-MRI rating was at least 2 months. Readers were allowed to assess all multiplanar reconstructions of the FRACTURE-MRI for decision-making.

Each reader first reviewed the images regarding the presence or absence of a proximal tibial fracture (yes/no). In the case of a positive fracture finding, all cases were classified according to the AO/OTA classification (types 41-A1 to 41-C3, (6)). Moreover, all AO/OTA type B and C fractures were classified according to Schatzker (I–VI, (7)) and the 10-segment classification (10).

#### Image quality

Image quality was rated as follows and as described previously [15, 16]: a five-point Likert scale was applied for image quality rating (5 = excellent definition of fracture line and fracture displacement, no influence on clinical decision-making; 4 = good definition of fracture line and fracture displacement, no influence on clinical decision-making; 3 = adequate definition of fracture line and fracture displacement, no influence on clinical decision-making; 2 = poor definition of fracture line and fracture displacement, impacts clinical decision-making; 1 = inadequate definition of fracture line and fracture displacement; impacts clinical decision-making). Image examples for the respective Likert scale ratings are provided in Supplementary Fig. 1.

#### Statistical analysis

Image quality of FRACTURE-MRI was assessed by calculating estimated marginal means with 95% confidence intervals (CI) using the factors *center* and *reader* as covariates.

The diagnostic performance of FRACTURE-MRI for the detection of proximal tibial fractures was assessed using crosstabulations. Patients with fractures other than in the proximal tibia were considered as having no fracture in the statistical analysis. An inconclusive result was given when the reader achieved a partially correct rating, i.e., detected the fracture but classified it incorrectly. According to the intention-to-diagnose principle [17], inconclusive results were considered false results, leading to a conservative estimation of diagnostic performance. Krippendorff’s alpha with bootstrapped 95% CI was calculated to estimate inter-reader agreement (0 = no agreement, 1 = perfect agreement) [18]. Mann–Whitney U-Test was performed to evaluate whether image quality differed between correctly versus wrongly diagnosed/classified fractures.

Statistical analysis was performed using MedCalc Statistical Software version 20.215 (MedCalc Software Ltd) and SPSS Version 27.0 (IBM Corp. IBM SPSS Statistics).

## Results

### Study population and reference standard

In total, 126 patients (*n*_center1_ = 58; *n*_center2_ = 68) were included in the study. Acute tibial fractures were present in *n* = 41 (32.5%) patients. Six patients had other fractures than in the proximal tibia (4 × isolated distal femur; 2 × isolated fibula). These patients were considered fracture-negative, as only fractures of the tibia were used for statistical analysis. Table 2 provides the descriptive statistics of the included study cohort including the classifications according to the AO/OTA and Schatzker classification. Using the 10-segment classification, CT identified 164 out of 410 possible segments as fractured, corresponding to a mean of 4.1 ± 2.2 segments per patient (range 1–9).

**Table 2:** Descriptive statistics of the study cohort

Characteristic	Frequency	
Included patients	126	
Age (years)	39.6 ± 14.5	
Sex	85 male, 41 female	
Presence of a proximal tibial fracture	41 (32.5%)	
Fracture classification according to		
AO/OTA	A1	2
	B1	8
	B2	13
	B3	8
	C3	10
Schatzker	I	5
	II	6
	III	14
	IV	4
	VI	10

### Image quality

The overall image quality of FRACTURE-MRI was rated good. The estimated marginal mean was 4.3 (95% CI: 4.1–4.4), with 43.6% (110/252) of cases rated as excellent (Likert 5), 43.6% (110/252) as good (Likert 4), 11.1% (28/252) as adequate (Likert 3), 1.6% (4/252) as poor (Likert 2), and none as inadequate (Likert 1). Inter-reader agreement was fair with Krippendorff’s alpha of 0.75 (95% CI: 0.66–0.83).

### Diagnostic performance

**Fig. 2:**
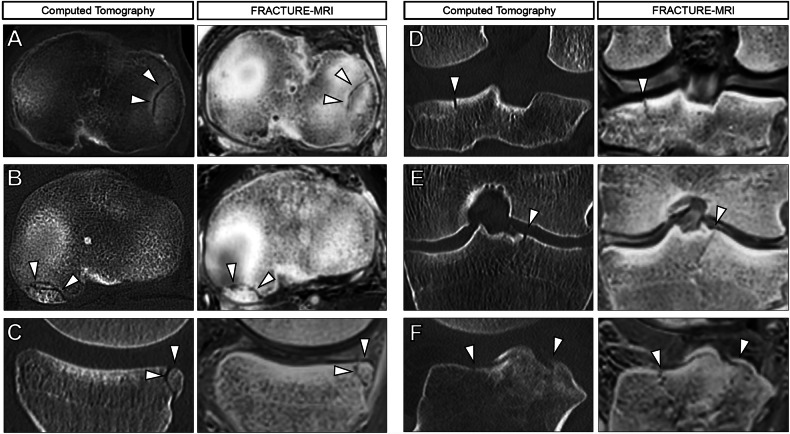
Comparison of exemplary nondisplaced tibial plateau fractures in computed tomography (CT, left columns) and FRACTURE-MRI (right columns). Arrowheads highlight fracture lines. **A** Axial images show a fracture line in the lateral tibial plateau in a 61-year-old male (AO/OTA B2, Schatzker III). **B**, **C** Axial and sagittal images show fracture lines in the dorsolateral tibial plateau in a 28-year-old male (AO/OTA B2, Schatzker III). **D** Coronal images show a nondisplaced fracture in a 38-year-old male (AO/OTA B3, Schatzker III), in a 56-year-old female (AO/OTA B1, Schatzker I) (**E**), as well as in a 43-year-old male (AO/OTA B3, Schatzker II) (**F**) (sagittal views). Fracture lines can be sharply delineated on FRACTURE-MRI

**Fig. 3:**
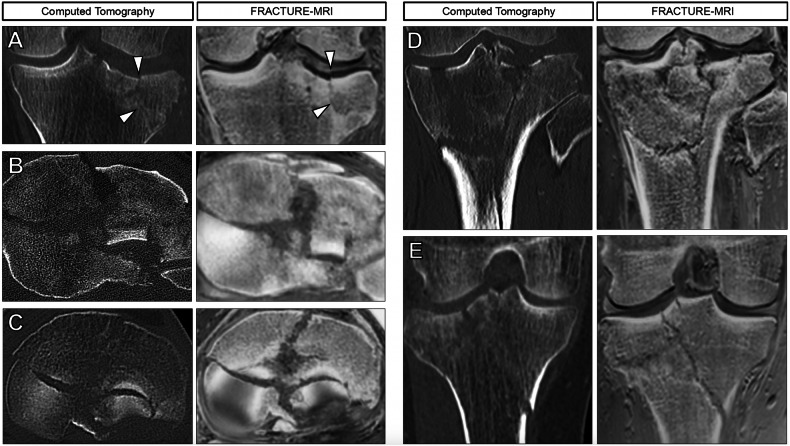
Comparison of exemplary displaced tibial plateau fractures in computed tomography (CT, left columns) and FRACTURE-MRI (right columns). Arrowheads highlight fracture lines. **A** Coronal images show an impression of the lateral tibial plateau with an associated fracture line in a 67-year-old female (AO/OTA B3, Schatzker II). **B** Axial images show displaced bicondylar fractures in a 61-year-old male (AO/OTA C3, Schatzker VI) and (**C**) in a 60-year-old male (AO/OTA C3, Schatzker VI). **D** Coronal images show displaced bicondylar fractures in a 61-year-old male (AO/OTA C3, Schatzker VI) (**E**) and a 25-year-old male (AO/OTA C3, Schatzker VI). Fracture lines are sharply delineated on FRACTURE-MRI

Figure 2 shows exemplary nondisplaced tibial plateau fractures of different patients in both CT and FRACTURE-MRI, while exemplary displaced tibial plateau fractures of different patients are visualized in Fig. 3. Fracture lines can be sharply delineated on FRACTURE-MRI, even in nondisplaced fractures.

Fractures were detected by both readers in 75 of 82 cases (41 fractures × 2 readers = 82). The number of false positives was *n* = 5 (*n*_reader1_ = 2; *n*_reader2_ = 3) and the number of false negatives was *n* = 7 (*n*_reader1_ = 4; *n*_reader2_ = 3). Sensitivity, specificity, positive and negative predictive value, and the accuracy of the FRACTURE-MRI for the different classifications and inter-reader agreement are provided in Table 3.

**Table 3:** Diagnostic performance and inter-reader agreement of FRACTURE-MRI

		Fracture detection*	AO/OTA^#^	Schatzker^#^	10-segment classification
Diagnostic performance	Sensitivity (%) (95% CI)	91.5 (83.2–96.5)	84.2 (74.4–91.3)	78.2 (67.4–86.8)	85.7 (81.4–89.3)
	Specificity (%) (95% CI)	97.1 (93.3–99.0)	97.1 (93.3–99.0)	97.7 (94.1–99.4)	97.4 (96.6–98.0)
	PPV (%) (95% CI)	93.8 (86.3–97.3)	93.2 (85.3–97.1)	93.9 (85.2–97.6)	82.9 (78.9–86.2)
	NPV (%) (95% CI)	95.9 (92.1–98.0)	92.7 (88.5–95.4)	90.7 (86.5–93.7)	97.9 (97.2–98.3)
	Accuracy	95.2 (91.8–97.5)	92.9 (89.0–95.7)	91.5 (87.4–94.7)	95.8 (95.0–96.6)
Inter-reader agreement (Krippendorff’s alpha)	Total data set (*n* = 126)	0.96 (0.91–1.0)	0.89 (0.79–0.96)	0.90 (0.79–0.98)	0.94 (0.91–0.96)
	Fractures only (*n* = 41)	0.85 (0.53–1.0)	0.48 (0.06–0.90)	0.69 (0.38–0.92)	0.91 (0.86–0.95)

*CI* confidence interval, *PPV* positive predictive value, *NPV* negative predictive value

* Binary: yes / no

^#^ According to the intention-to-diagnose principle, partially correct answers were considered false

According to the AO/OTA classification, fractures were correctly classified by both readers in 71 of 82 cases (86.6%; false cases *n*_reader1_ = 7; *n*_reader2_ = 4). Among the false cases, 4 were misclassified according to AO/OTA (1 × B1 instead of B2; 1 × B1 instead of B3; 1 × B3 instead of B1; 2 × B3 instead of B2; 1 × C2 instead of B1), and in 7 cases fractures were not detected at all (2 × B1; 5 × B2). The number of false positives was *n* = 5 (1 × A2; 4 × B2).

The AO/OTA type B and C fractures were correctly classified according to the Schatzker classification by both readers in 61 of 78 cases (78.2%; false cases *n*_reader1_ = 11; *n*_reader2_ = 6). Among the false cases, 8 were misclassified according to Schatzker (2 × I instead of III; 1 × I instead of IV; 2 × II instead of III; 2 × II instead of IV; 1 × IV instead of V), and in 9 cases fractures were not detected at all (2 × I; 7 × III). The number of false positives was *n* = 4 (4 × III).

According to the 10-segment classification, the sum of correctly classified segments by both readers was 281/328 (86.0%; 164 segments × 2 readers = 328). Forty-seven segments were falsely classified as intact (*n*_reader1_ = 23; *n*_reader2_ = 24), and 58 segments were falsely identified as fractured (*n*_reader1_ = 30; *n*_reader2_ = 28).

### Impact of FRACTURE-MRI image quality on fracture diagnosis and classification

Mann–Whitney U-Test revealed no statistical difference in image quality (Likert 1-5) between correctly versus wrongly diagnosed fractures (*z* = −1.20, *p* = 0.23). Regarding fracture classification, there was no statistical difference in image quality (Likert 1-5) between correctly versus wrongly classified fractures according to AO (*z* = −1.89, *p* = 0.06), to Schatzker (*z* = −0.97, *p* = 0.33), and the 10-segment classification (*z* = −1.19, *p* = 0.24).

## Discussion

This study evaluated the diagnostic performance of a fast field echo resembling a CT using restricted echo-spacing (FRACTURE) MRI sequence in patients following acute knee injury for the detection and classification of proximal tibial fractures in a representative patient collective. Distribution of the different fracture classes was comparable to previous studies and therefore representative. For example, Wennergren et al reported that type B fractures accounted for 64% of proximal tibial fractures, while A and C fractures only accounted for 18% each [19].

Overall, we found a substantial to perfect diagnostic performance for fracture detection (sensitivity 91.5% (83.2–96.5%); specificity 97.1% (93.3–99.0%)). Moreover, it can be hypothesized that the specificity of FRACTURE-MRI would have been even higher if fluid-sensitive sequences had been included in the assessment.

Regarding fracture classification, the diagnostic performance of FRACTURE-MRI was slightly lower than the detection rate, with the 10-segment classification yielding the best, and the Schatzker classification yielding the lowest sensitivity. The decrease in fracture classification performance is, at least in part, explicable by the conservative statistical estimation where detected but falsely classified fractures were counted as false cases. Regarding the differences in diagnostic performance of the evaluated classification systems, an explanation for the better performance of the 10-segment classification is that while this classification allows for precise description regarding the anatomical topography of fracture fragments, hereby allowing little room for discussion, it fails to adequately characterize fracture morphology and displacement (split and/or depression) which is taken into account by the Schatzker and AO classification [6]. The latter of which may require more user experience and consequently result in a higher rate of false classifications.

Inter-reader agreement across the whole cohort was excellent (Krippendorff’s alpha 0.89–0.96), and when considering only patients with fractures, acceptable (0.48–0.91), i.e., comparable to what was reported for CT. For example, inter-reader agreement on three-dimensional CT imaging of proximal tibial fractures varies considerably, ranging from fair to moderate to good with kappa values of 0.36–0.76 for the Schatzker classification [20–25], 0.23–0.83 for the OA/OTA classification [21–25], and only 0.27 for the 10-segment classification [25]. In summary, a clear picture of the value of the different classifications does not emerge. For example, Maripuri et al concluded that none of the classification systems are ideal, however, the Schatzker classification was superior to the AO/OTA classification both in terms of inter- and intra-observer agreement [26].

Adding to this, it has been shown that reliability estimates for proximal tibial fracture classification can be improved by including multiple slices, i.e., by using three- instead of only two-dimensional scans [6, 24]. In line with these considerations, the proposed 3D FRACTURE-MRI sequence enables the reconstruction of multiplanar reformations for assessment of often highly variably and complex fracture patterns as required for precise preoperative visualization and surgical treatment planning.

For patients requiring a preoperative MRI anyway as well as from an economic perspective, it would be desirable to acquire all information using one cross-sectional imaging modality instead of two. FRACTURE-MRI might therefore serve as an alternative to a conventional computed tomography scan in an acute trauma setting by reducing both costs and radiation exposure.

Among existing bone imaging techniques in MR imaging, FRACTURE, in general, benefits from high resolution (sub-millimeter isotropic), easy implementation, ready compatibility to any imaging acceleration technology, including all vendors’ versions of compressed sensing for further scan time reduction and wide clinical adaption [12, 13], compared to, for example, UTE or ZTE (zero TE) that requires both dedicated sequence and sophisticated reconstruction [27, 28]. On the other hand, the indirect representation of the FRACTURE images, exploring inverted magnitude images, is not common practice for radiology interpretation, though a similar concept has been elsewhere reported [29]. Previous studies have used FRACTURE-MRI to visualize osseous structures [15, 27, 28, 30]. This study is, to our knowledge, the first systematically investigating the clinical performance of the presented 3D fast field echo resembling a CT using restricted echo-spacing MRI technique in the knee compared with standard CT.

The study had the following limitations: First, CT was only performed in patients with acute fractures, serving as the reference standard, which is explained by the retrospective study design, as according to the clinical workflow, it was not indicated in patients without fractures. However, the exclusion of significant acute trauma of the skeletal structures of the knee based on the evaluation of the conventional radiographs and the whole MRI protocol, including fluid-sensitive sequences, seems fair.

Second, only patients with acute fractures were included. Future studies must clarify whether FRACTURE-MRI is equally applicable for the evaluation of pathological fractures or bone metastases.

Third, we exclusively investigated the diagnostic performance of FRACTURE-MRI for the assessment of proximal tibial fractures. Future studies need to address in a comparative intra-individual setting whether FRACTURE-MRI performance is better or equivalent to other CT-like bone imaging techniques, such as UTE/ZTE, GRE, or SWI, for fracture imaging. The present study did not compare other CT-like MRI techniques, such as UTE or SWI [12, 13], which is explained by the clinical setting due to maximum exam time and patient compliance. A literature review has been provided previously by Lombardi and Chong [12, 13], and an intra-individual comparison may be considered in future studies.

The susceptibility to metallic artifacts is another limitation of all CT-like MRI sequences in patients with metallic implants or those who underwent knee-spanning external fixation. The feasibility of FRACTURE-MRI and/or other CT-like MRI sequences must be evaluated in further studies. For example, a preoperative CT is often acquired to allow for intra-individual comparison with the postoperative result regarding fracture reduction and consolidation.

Last, it should be considered that MRI is less accessible than CT in emergency settings, as it requires a longer examination time and greater patient compliance. Therefore, the application of FRACTURE-MRI may be limited to centers with the necessary resources and/or those patients who would otherwise also undergo MRI in addition to CT. In this scenario, FRACTURE-MRI could simplify diagnostic imaging and thus save resources and costs.

In summary, FRACTURE-MRI is feasible at 3 T and allows reliable delineation of fracture lines for precise diagnosis and classification of proximal tibial fractures. FRACTURE-MRI might therefore serve as a helpful alternative to computed tomography in an acute trauma setting by reducing both costs and radiation exposure in patients requiring a preoperative MRI anyway.

## Supplementary information

ELECTRONIC SUPPLEMENTARY MATERIAL

## Abbreviations

AO: Arbeitsgemeinschaft für Osteosynthesefragen (Foundation for classifying bone fractures)
CI: Confidence interval
CT: Computed tomography
FRACTURE-MRI: Fast field echo resembling a CT using restricted echo-spacing
GRE: Gradient echo
MRI: Magnetic resonance imaging
SWI: Susceptibility-weighted imaging
UTE: Ultra-short echo time
ZTE: Zero echo time
