# Supplementary material for: Assessment of proximal tibial fractures with 3D FRACTURE (fast field echo resembling a CT using restricted echo-spacing) MRI—intra-individual comparison with CT
Source: Eur Radiol. 2025 Mar 24;35(9):5418–26. doi: 10.1007/s00330-025-11522-3 (PMC12350421; doi:10.1007/s00330-025-11522-3)
Supplement: Supplementary file 1 — ELECTRONIC SUPPLEMENTARY MATERIAL [file 330_2025_11522_MOESM1_ESM.pdf]

Assessment of proximal tibial fractures with 3D FRACTURE (fast field echo resembling a CT using restricted echo-spacing) MRI – Intraindividual comparison with CT

ELECTRONIC SUPPLEMENTARY MATERIAL

Eur Radiol (2025) Ristow I, Zhang S, Riedel C, et al.

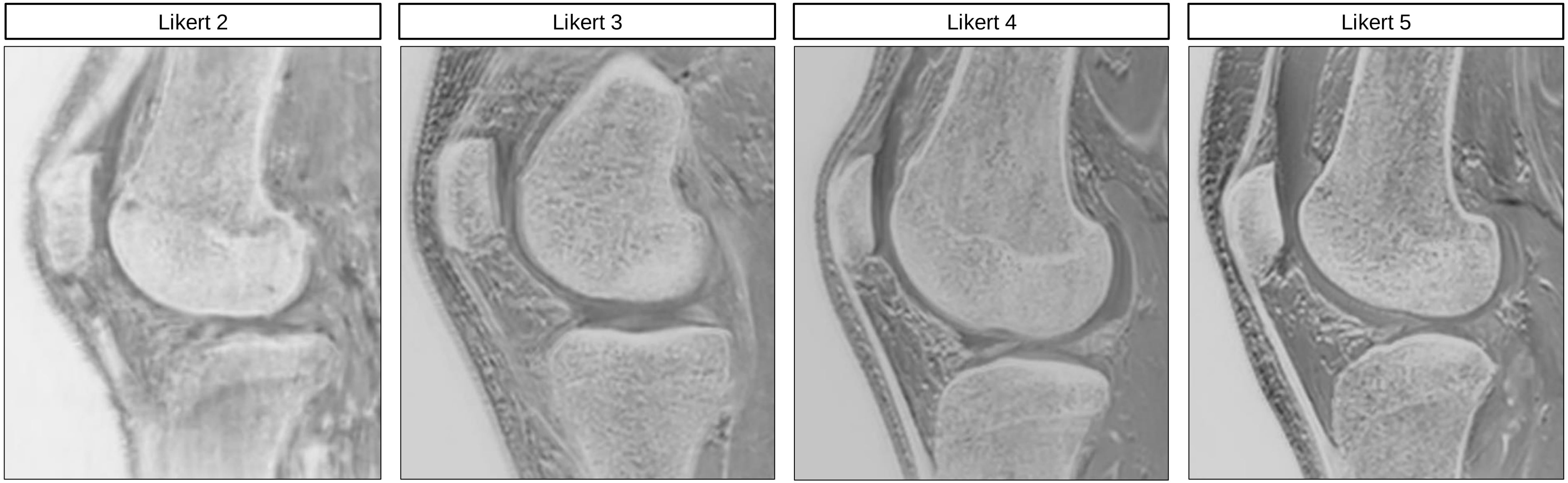

Supplementary Figure 1: Examples for image quality ratings Likert 2 – 4.

No MRI scan was rated Likert 1 by both readers. Note the motion-related artifacts in Likert 2 and 3, resulting in cortical blurring.

Likert 1 = inadequate definition of fracture line and fracture displacement; impacts clinical decision-making

Likert 2 = poor definition of fracture line and fracture displacement, impacts clinical decision-making

Likert 3 = adequate definition of fracture line and fracture displacement, no influence on clinical decision-making

Likert 4 = good definition of fracture line and fracture displacement, no influence on clinical decision-making

Likert 5 = excellent definition of fracture line and fracture displacement, no influence on clinical decision-making
